# The Role of TAL1 in Hematopoiesis and Leukemogenesis

**Published:** 2018

**Authors:** E. R. Vagapova, P. V. Spirin, T. D. Lebedev, V. S. Prassolov

**Affiliations:** The Engelhardt Institute of Molecular Biology, Russian Academy of Sciences, Vavilova Str. 32, Moscow,119991, Russia

**Keywords:** hematopoiesis, acute myeloid leukemia, receptor tyrosine kinase C-KIT, T-cell acute lymphoblastic leukemia

## Abstract

TAL1 (SCL/TAL1, T-cell acute leukemia protein 1) is a transcription factor that is involved in the process of hematopoiesis and leukemogenesis. It participates in blood cell formation, forms mesoderm in early embryogenesis, and regulates hematopoiesis in adult organisms. TAL1 is essential in maintaining the multipotency of hematopoietic stem cells (HSC) and keeping them in quiescence (stage G0). TAL1 forms complexes with various transcription factors, regulating hematopoiesis (E2A/HEB, GATA1–3, LMO1–2, Ldb1, ETO_2_, RUNX1, ERG, FLI1). In these complexes, TAL1 regulates normal myeloid differentiation, controls the proliferation of erythroid progenitors, and determines the choice of the direction of HSC differentiation. The transcription factors TAL1, E2A, GATA1 (or GATA2), LMO2, and Ldb1 are the major components of the SCL complex. In addition to normal hematopoiesis, this complex may also be involved in the process of blood cell malignant transformation. Upregulation of *C-KIT *expression is one of the main roles played by the SCL complex. Today, TAL1 and its partners are considered promising therapeutic targets in the treatment of T-cell acute lymphoblastic leukemia.

## INTRODUCTION

Hematopoiesis comprises a series of steps, including the formation of early hematopoietic progenitor cells from mesoderm, the formation of hematopoietic stem cells (HSC), and their further differentiation into mature blood cells. Dysregulation of these processes in hematopoietic precursor cells often leads to their abnormal differentiation and proliferation and, as a result, malignant transformation. The transcription factor TAL1 is one of the main regulators of hematopoiesis. It comprises a helix-loop-helix domain which binds to DNA through its regulatory regions, interacting with the E-box sequence (CANNTG, where N is any nucleotide), and GATA, Ets, and Runx factor binding sites [1]. It has been shown that inhibition of *TAL1 *gene expression leads to a complete absence of hematopoiesis in the yolk sac [2]. In an adult organism, a mximum level of *TAL1 *expression is characteristic of pluripotent HSCs, multipotent myeloid and lymphoid progenitors, as well as erythroid and megakaryocytic cells [3]. TAL1 participates in the formation of complexes with various transcription factors (E47/E2A, LMO2, GATA1–3, Ldb1/2, Ldb1, ETO_2_, Runx1, ERG, FLI1) [4, 5]. The composition of the complex may vary. The composition of the complex determines the intracellular targets it interacts with, activating or inhibiting the expression of the factors associated with differentiation of myeloid and lymphoid cells [6-8]. An abnormal expression level or mutations in genes whose translation products comprise the SCL complex can lead to malignant transformation of blood cells. Approximately 60% of cases of T-cell acute lymphoblastic leukemia (T-ALL) are characterized by an abnormally high level of *TAL1 *expression [9]. Mutant forms of TAL1 in lymphoid and myeloid leukemia cells are diagnosed in 20% of patients [10]. The promoter portion of the *C-KIT *gene encoding the receptor tyrosine kinase is considered as one of the main TAL1 targets in malignant blood cells. In some cases, it has been shown that progression of malignant hematological diseases (including acute myeloid leukemia) is accompanied by an abnormally high expression of C-KIT [11, 12].

## TAL1: GENE STRUCTURE, KNOWN ISOFORMS OF THE PROTEIN AND THEIR FUNCTION IN HEMATOPOIESIS

**Fig. 1:**
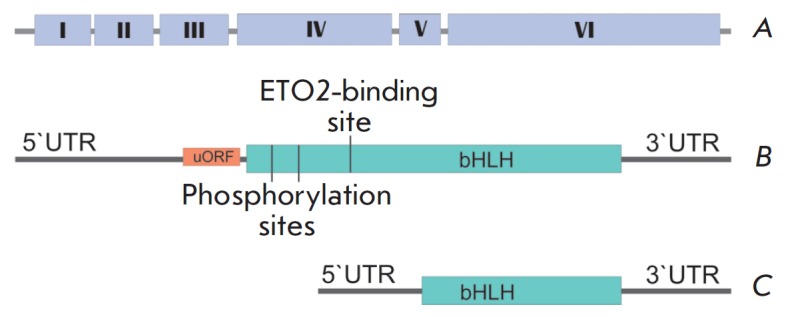
a. The structure of TAL1 gene – I–VI exons b. Long TAL1 transcript variant. Long isoform TAL1-L and short isoform TAL1-S are translated from this mRNA. UTR – untranslated mRNA region. uORF – upstream open reading frame. bHLH – mRNA region encoding the helixloop- helix domain. c. Short TAL1 transcript variant from which only the TAL1-S isoform is translated

The *TAL1 *gene locus is located on human chromosome 1. TAL1 belongs to the family of transcription factors that possess a helix-loop-helix (bHLH) motif. The *TAL1 *gene contains six exons, including the coding exons 4–6. According to the PubMed database as of 2017, six different transcripts of the *TAL1 *gene have been described (*Fig. 1*). There are two isoforms to the TAL1 protein: a long (TAL1-l) one, with a molecular weight of 34.3 kDa and composed of 331 amino acid residues, and a short (TAL1-s) one, consisting of 156 amino acid residues. The TAL1-l to TAL1-s ratio differs in megacaryocyte-erythroid cells [13]. *TAL1 *pre-mRNA is alternatively spliced, producing mRNA without the exons 1–4. The ETO_2_-binding domain and phosphorylation sites are absent in the TAL1-s protein translation product of this mRNA, while DNA-binding domains and the helix-loop-helix domain are maintained. Furthermore, the third exon of the *TAL1 *comprises a highly conserved uORF sequence, an upstream open reading frame which acts as a *cis-*regulatory element in the formation of TAL1 isoforms. The presence of uORF enables the initiation of translation, involving the eIF2 and eIF4E factors from the alternative sites located in exons 4–5 [14], producing a truncated form of the TAL1 protein.

The truncated form TAL1-s is required for erythroid progenitors differentiation, while the full-length protein TAL1-l is required for megakaryocytic differentiation of progenitor cells. It has been shown that treatment of the human erythroid leukemia cell lines TF1 and HEL with erythroid differentiation inducers (DMSO and erythropoietin) produces not only the primary (full-length) form of the TAL1-l protein, but also a truncated TAL1-s form [15]. It has been established that some anticancer agents acting on the components of the signaling pathways involved in the regulation of translation initiation may affect the TAL1-l to TAL1-s ratio. In particular, rapamycin (Rap,mTOR inhibitor) blocks the formation of truncated forms, while 2-Aminopurine (2AP, eIF2α-kinase inhibitor) blocks the formation of full-length forms [14].

## TAL1 FUNCTIONS IN EMBRYOGENESIS

The TAL1 transcription factor is essential for normal embryogenesis. Its expression starts on the 7^th^ day after fertilization, a day before the beginning of the development of circulatory system components. *TAL1 *expression has been found in the blood islet cells of the yolk sac, endothelial cells, and angioblasts, and then in the liver and spleen of a fetus, the major hematopoietic organs in embryogenesis. It has been shown that the cells involved in the formation of skeletal and nervous tissues also express *TAL1 *[16]. In the yolk sac and fetal liver, the *Runx1 *gene promoter and *Runx3 *gene enhancer are the major targets of TAL1 [17]. Ets, GATA, and the Runx factor binding sites, as well as a E-box sequence, have been found in the regulatory regions of these genes. TAL1 and its partners GATA1, GATA2, E47, Ldb1, and LMO2 may form complexes at these DNA sites [18]. Hematopoietic progenitor cells can also be derived from hemogenic endothelial cells, a process that involves the Runx1 transcription factor. TAL1 is required in order to produce hemogenic endothelial cells from mesoderm [19]. At later stages of embryonic development, TAL1 regulates the differentiation of hematopoietic progenitors into red blood cells, megakaryocytes, and platelets [20]. During embryogenesis, the cells that form blood vessels also express *TAL1 *[16]. A lack of *TAL1 *expression not only results in impaired hematopoiesis, but also in early embryonic death [2, 21]. It has been demonstrated in a murine model that embryonic stem cells (ESCs) not expressing *TAL1 *are not differentiated into hematopoietic cells under the action of hematopoietic differentiation factors [21]. Ectopic expression of *TAL1 *in ESCs induces the formation of hematopoietic cells. *In vitro *experiments have demonstrated that ESCs without *TAL1 *expression are characterized by a low effectiveness of differentiation into erythroid progenitor cells and cannot form colonies of lymphoid and myeloid progenitor cells [22].

**Fig. 2:**
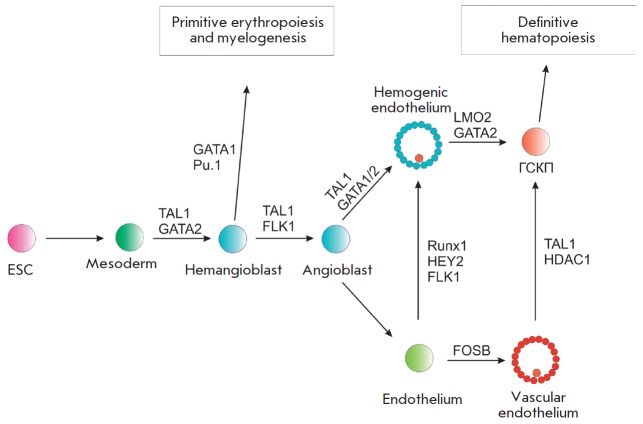
The process of hematopoietic cell development during embryogenesis. Some transcription factors defining the differentiation direction are shown above the arrows. ESC – embryonic stem cell, HSCP – hematopoietic stem cell progenitor

Thus, TAL1 directs the differentiation of hematopoietic progenitors at all three stages of hematopoiesis during embryonic development. TAL1 acts on blood progenitor cells in the yolk sac (the first stage of hematopoiesis), determines the development and differentiation of hemangioblasts from their aggregation in the primary strip until their migration into the hematopoietic islets of the yolk sac (the second stage of hematopoiesis). At the beginning of the third stage of hematopoiesis, TAL1 is required for hemangioblast differentiation in HSCs. It activates the expression of genes important for the maturation of erythroid, megacaryotic, and mast cells, and it is likewise involved in vascular system remodeling (*Fig. 2*) [23].

## THE ROLE OF TAL1 IN THE REGULATION OF HEMATOPOIESIS

**Fig. 3:**
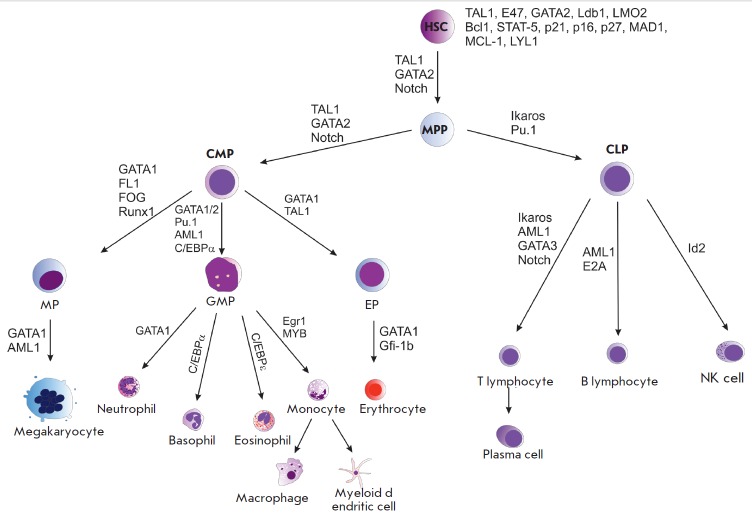
Adult hematopoiesis scheme with some key transcriptional factors. HSC – hematopoietic stem cell, MPP – multipotential progenitor, CMP – common myeloid progenitor, CLP – common lymphoid progenitor, MP – megakaryocyte progenitor, EP – erythropoietin progenitor, EBP – eosinophil–basophil progenitor

In adults, mature blood cells are derived from pluripotent HSCs. HSCs are retained in the bone marrow during replication quiescence stage G0, due to the interaction between their superficial cellular receptor protein (C-KIT, MPL, CXCR4) and the ligands on stromal cell surfaces [24, 25]. The pluripotent HSCs respond to hematopoietic stress by terminating the quiescent phase and initiating active proliferation, receiving signals for further differentiation, and leading to the appearance of myeloid and lymphoid progenitor cells. Some transcription factors essential to the hematopoiesis process are also the key factors in maintaining HSCs in the quiescent stage. These include the TAL1, E47, GATA2, and Ldb1, LMO2 components of the SCL complex [26]. Transformation of KLS+/CD150+/CD48+ HSCs from the quiescent phase G0 to stage G1 is assisted by the cyclin-dependent kinase P21/CDKN1A. TAL1 blocks this transition, increasing the expression of the P21/ CDKN1A inhibitor [27]. Simultaneously, TAL1 enhances the expression of the transcription factor ID1. Importantly, TAL1 does not belong to the proteins essential for the survival and self-renewal of HSCs [3]. Its related protein LYL1 supports HSC survival in the case of *TAL1 *knockout [28]. Interestingly, TAL1 plays the opposite role in cord blood HSCs, where it, contrarily, activates G0–G1 transition, which is regulated using the mTOR signaling pathway [29]. However, TAL1 and LYL1 are not interchangeable in differentiation processes and both proteins are required for normal erythropoiesis and the formation of B-cells, respectively [30]. Unlike HSCs, TAL1 functions as a cell cycle activator in myeloid and lymphoid progenitors, inhibiting the expression of the cyclin-dependent kinase inhibitors p21 and p16/Ink4a [31, 32]. The hematopoietic transcription factors TAL1, GATA2, and LMO2, whose expression level differs in each cell type, regulate the process of blood cell differentiation and maturation *(Fig. 3)* [33]. The expression of *TAL1 *is not identical in all hematopoietic cells. High expression levels of this gene have been detected in HSCs, in myeloid progenitors, and in some mature myeloid cells (megakaryocytes, erythrocytes, mast cells, and basophils). Low levels of TAL1 are characteristic of lymphoid progenitors, eosinophils, macrophages, and neutrophils [34-36]. Mature T- and B-cells do not express *TAL1* [37]. Certain genes specific to erythroid cells are activated by a complex formed by GATA1 and TAL1 [38].

An analysis of the ChIP-seq has shown that TAL1 controls both the processes common to all cells (cell cycle regulation, proliferation, apoptosis) and those specific only to erythroid cells (redox processes, heme biosynthesis, organization of the cytoskeleton), which is indirectly indicative of its multifunctionality [39]. In myeloid and lymphoid progenitor cells, the genes that control proliferation and apoptosis play the role of TAL1 targets. Additionally, the pattern of TAL1 binding to target genes widely varies with cell maturation. The dynamic changes in *TAL1 *expression suggest that the TAL1 factor demonstrates differing activity in cells during the initial choice of differentiation direction and formation of mature blood cells, while its multifunctionality is directly related to its ability to form multicomponent complexes in the regulatory regions of target genes [8]. There is evidence that the role of TAL1 in the differentiation of erythroid cells is effected, among others, using caspase-3, inducing cleavage of this protein. It has been shown that its activity eventually leads to a decrease in the expression of *GATA1* and *BCL-XL, *thereby inducing apoptosis in these cells [40]. Some amino acid residues of TAL1 may undergo phosphorylation. For example, in erythrocytes, Akt kinase phosphorylates Thr 90 in TAL1. This modification reduces the ability of TAL1 to repress the *EPB42 *gene promoter, whose product, the 4.2 protein, is required to build the erythrocyte cytoskeleton [41]. The Ser172 residue may also be phosphorylated by the cAMP-dependent protein kinase (PKA), which affects TAL1 binding to the E-box in the regulatory sites of various genes [42].

## SCL-COMPLEX: ITS COMPONENTS AND TARGETS IN NORMAL HEMOPOIESIS

**Fig. 4:**
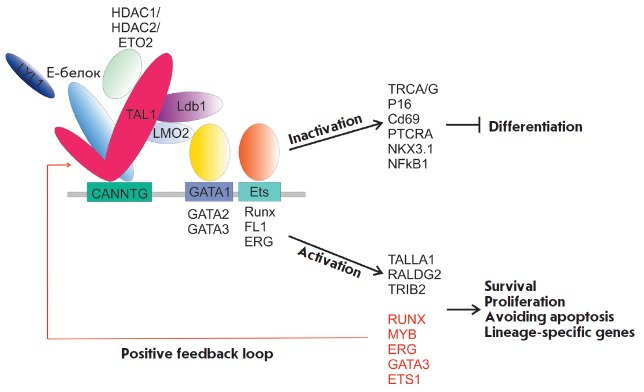
TAL1 and its partners involved in the differentiation, proliferation, and survival of hematopoietic cells

The proteins involved in normal hematopoiesis (LMO2, Ldb1–2, Gata1–3, Lyl–1, E2A/HEB, Runx1, ETO2, ERG, FL1) are the main partners of TAL1 in hematopoietic cells (*Fig. 4*). TAL1 directly binds to the LIM-domain of the LMO2 protein, which, in turn, interacts with Ldb1. LMO2 has no DNA-binding domain and acts as a bridge factor, which complexes TAL1 with other transcription factors in hematopoietic cells [43, 44]. It may also form an extended complex, binding ETO2, RUNX1, ERG, or FLI1 [45]. E-proteins (E12, E47), containing helix-loop-helix domains, are required for TAL1 binding to the E-box sequences (CANNTG) in the regulatory regions of genomic DNA. In the complex, TAL1 regulates the activity of certain signaling pathways during the differentiation of hematopoietic cells. For example, TAL1 is essential for the survival of hematopoietic precursors cultured in the presence of SCF, a ligand of the receptor tyrosine kinase C-KIT, which plays an important role in hematopoiesis [46]. The main role of the SCL complex in C-KIT regulation is associated with its ability to bind the promoter of this gene. It has also been established thatcomponents of the SCL complex may bind to various components of the C-KIT signaling pathway and change its activity [46-51].

Furthermore, there is a direct correlation between the level of *TAL1 *expression and phosphorylated forms of MEK and ERK1/2 kinases, the components of the MEK/ERK signaling pathway [40]. In hematopoietic cells, the activity of MEK and ERK1/2 kinases is associated with the differentiation of myeloid, erythroid, and megakaryocytic hematopoietic cells [52]. TAL1 probably participates in the differentiation of CD34+ hematopoietic cells through the MEK/ERK signals [52, 53].

## THE FUNCTIONS OF THE SCL COMPLEX AND ITS INDIVIDUAL COMPONENTS IN CARCINOGENESIS

As noted above, the normal level of *TAL1 *expression in lymphoid cells is much lower than that in myeloid ones [37]. Enhanced expression of *TAL1 *in T-cells often leads to their malignant transformation. Abnormally high expression of *TAL1 *can result from chromosomal rearrangements, deletions, and mutations affecting the gene [54]. The chromosomal translocation t (1; 14) (p32; q11) was found in 3% of cases of T-cell leukemia. The chromosomal translocation t (1; 14) (p32; q11), leading to the formation of the TRA/TAL1 fusion gene, was detected in 3% of cases of T-cell leukemia. Deletion of 90 bps between the 5`-noncoding region of the *TAL1* gene and *SIL *gene results in the formation of a *SILTAL1* fusion gene controlled by the *SIL *gene promoter [54]. The expression level of *SIL *in T-cells is normally very high, and, therefore, this translocation results in a high expression of the *SIL-TAL1 *fusion gene [55]. This deletion has been detected in 20–25% of patients with T-ALL [54, 56, 57]. However, in most TAL1-positive cases of T-cell leukemia, an abnormally high expression of *TAL1 *is effected without the participation of chromosomal rearrangements. Along with a high expression of *TAL1*, significant expression levels of *TLX1* and *LMO2*were detected in most primary T-ALL samples [58]. Increased activity of *TAL1 *in T-cells results in an increased lifetime for lymphoid cells in the form of immature thymocytes. It is assumed that this can be considered as an event initiating the development of T-cell leukemia [59].

In T-ALL cells, TAL1 preferably binds to CAGGTG E-box sequences. Although GATA1–3 factors often serve as intermediaries in TAL1 binding to the regulatory sites of DNA in T-cell leukemia cells, there are alternative binding sites, in particular Runx and Ets [59]. It has been shown that the TAL1 transcription factor directly activates the expression of *Runx1*, *Ets1, *and* GATA3 *in the blast cells of patients with T-ALL [60]. Furthermore, the GATA3 and Runx1 factors enhance the expression of the *TAL1 *gene, which may indicate the need for a positive feedback loop for the abnormal expression of the factors involved in blood cell malignant transformation. In 45% of cases of TAL1-positive leukemia, LMO1 and LMO2 mutant proteins formed due to chromosomal rearrangements of their encoding genes were detected [61]. Expression of all these factors leads to the fact that double negative (CD4-CD8-) preleukemic thymocytes become capable of division. Additionally, the Notch signaling pathway, whose components are involved in the accumulation of mutations and impairment of differentiation processes, is often activated in these cells. This leads to initiation and progression of T-cell leukemia [62]. In the case of malignant transformation , TAL1 is often involved in the abnormal transcription of various genes. In this case, as in normal hematopoiesis, it forms complexes with the hematopoietic factors LMO2, Ldb1, and E12/E47 [46,47]. It has been established that overexpression of TAL1 and LMO2 is often observed in T-ALL cells. Normally, LMO2 and TAL1 independently regulate the transcription of their own target genes, but they cooperatively disrupt the functioning of the E2A factor in T-ALL cells, which contributes to the development of leukemia [63, 64]. It has been shown that the transcription factor FOXP3 can act as a tumor suppressor in T-cell leukemia. It binds to LMO2 and reduces the likelihood of it interacting with TAL1, resulting in reduced transcriptional activity of the TAL1/LMO2 complex [65].

**Fig. 5:**
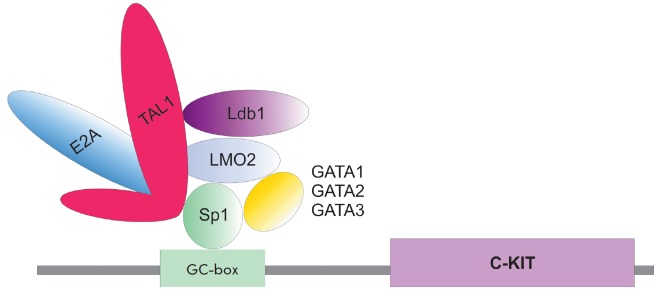
The structure of the SCL complex in the promoter region of the receptor tyrosine kinase C*-KIT *gene

C-KIT receptor tyrosine kinase is one of the main targets of TAL1 [48, 66]. Hematopoietic progenitor cells are characterized by a high expression level of *TAL1* and *C-KIT. *It has been shown that ectopic expression of *TAL1 *results in the induction of *C-KIT *expression in B-lymphocytes, which normally do not express these genes [66]. Some hematological malignancies, including acute myeloid leukemia and chronic myeloid leukemia, are associated with an abnormally high expression of* C-KIT. *The SCL complex acts as a specific activator receptor tyrosine kinase *C-KIT *gene promoter (*Fig. 5*). All the components of the complex (TAL1, LMO2, Ldb1, GATA2, E47) are required for it to function at its maximum. Studies in a murine embryonic fibroblast model have shown that the transcription factors E47 and GATA, taken alone, do not affect the activity of the *C-KIT *gene promoter despite the fact that they activate the transcription of many genes in human hematopoietic cells [66]. The same murine system was used to show that the promoter is only activated in the case of formation of a multicomponent complex whose main component is TAL1. GATA1 and GATA2 are interchangeable: however, the complex comprising GATA1 possess a lower transcriptional activity. The Sp1 protein, comprising zinc fingers and binding GC-rich sequences, is also required to form the active SCL-protein complex. It has been shown that removal of E-box and GATA from the promoter region of* C-KIT *does not reduce the activating activity of the SCL complex. Probably, Sp1 is also involved in attracting complex components to certain target genes.

## CLINICAL SIGNIFICANCE OF TAL1

**Fig. 6:**
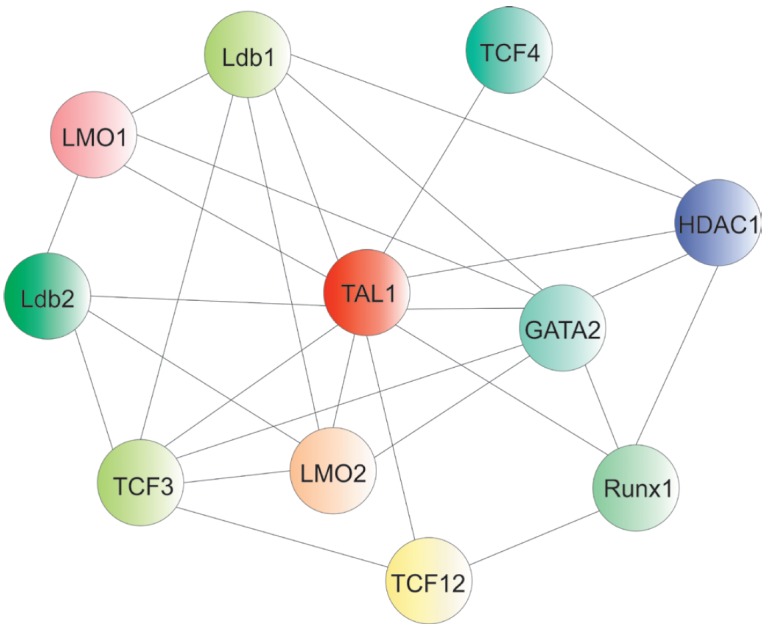
Possible partners of TAL1 and their interactions in normal and malignant hematopoietic cells

The extensive body of evidence of TAL1 participation in the development of T-cell leukemia suggests that inhibitors of this protein, as well as inhibitors of the associated signaling cascades, can be used as promising therapeutic agents to treat leukemia characterized by an abnormal activity of TAL1. At the moment, novel low-molecular-weight inhibitors of TAL1 are being developed and synthesized in many laboratories. However, sufficiently strong and specific inhibitors of this protein have not been achieved so far. Phosphorylation of TAL1 with MEK/ERK kinases is required to effect its transcriptional activity. The prospects of using the inhibitors of MAPK/MEK/ERK signaling pathway components as potential therapeutic targets are being discussed [67]. At the same time, there is evidence that treatment of a mesenchymal stromal cell culture (stromal components of the bone marrow) with MEK inhibitors results in the secretion of proinflammatory cytokine interleukin-18 by these cells [68]. This improves the survival chances of T-ALL blast cells. The potential TAL1 protein targets associated with the implementation of its transcriptional activity are considered as promising targets for the therapy of TAL1-associated T-cell leukemia (*Fig. 6*). These proteins include UTX demethylase (also known as KDM6A). It has been shown that treatment of TAL1-positive blast cells with the T-ALL UTX inhibitor reduces the rate of their proliferation and stimulates apoptosis [69]. It has been determined that the use of HDAC histone deacetylase inhibitors leads to a decrease in *TAL1* expression and induces the apoptosis of blast cells of T-cell leukemia [70]. At the moment, the stoichiometry of the SCL complex is being actively explored. The results of such studies are expected to open up new possibilities for the development of highly effective therapeutic agents targeting TAL1-positive leukemia, which could act by interfering with the protein-protein interactions between the components of the SCL complex but not affect the viability of normal hematopoietic cells [41].

## Abbreviations

HSC: hematopoietic stem cell
ESC: embryonic stem cell
CMP: common myeloid progenitor
CLP: common lymphoid progenitor
T-ALL: T-cell acute lymphoblastic leukemia
DMSO: dimethyl sulfoxide
